# Comparative occurrence of diabetes in canine, feline, and few wild animals and their association with pancreatic diseases and ketoacidosis with therapeutic approach

**DOI:** 10.14202/vetworld.2018.410-422

**Published:** 2018-04-05

**Authors:** Kamal Niaz, Faheem Maqbool, Fazlullah Khan, Fatima Ismail Hassan, Saeideh Momtaz, Mohammad Abdollahi

**Affiliations:** 1 Department of Toxicology and Pharmacology, International Campus, Tehran University of Medical Sciences, Tehran, Iran; 2 The Institute of Pharmaceutical Sciences, Tehran University of Medical Sciences, Tehran, Iran; 3 Medicinal Plants Research Center, Institute of Medicinal Plants, ACECR, Karaj, Iran; 4 Department of Toxicology and Pharmacology, Faculty of Pharmacy, Tehran University of Medical Sciences, Tehran, Iran

**Keywords:** amyloidosis, diabetes mellitus, gestational diabetes mellitus, hypercortisolism, necrosis, pancreatitis

## Abstract

Diabetes mellitus (DM) is a chronic metabolic disorder in which blood glucose level raises that can result in severe complications. However, the incidence increased mostly by obesity, pregnancy, persistent corpus luteum, and diestrus phase in humans and animals. This review has focused on addressing the possible understanding and pathogenesis of spontaneous DM in canine, feline, and few wild animals. Furthermore, pancreatic associated disorders, diabetic ketoacidosis, hormonal and drug interaction with diabetes, and herbal remedies associated with DM are elucidated. Bibliographic search for the present review was done using PubMed, Scopus, and Google Scholar for articles on concurrent DM in small and wild animals. Persistent corpus luteal and pseudopregnancy in female dogs generate gestational DM (GDM). GDM can also be caused by extensive use of drugs/hormones such as glucocorticosteroids. Although many similarities are present between diabetic cats and diabetic humans which present islet amyloidosis, there was a progressive loss of β- and α-cells and the normal number of δ-cells. The most prominent similarity is the occurrence of islet amyloidosis in all cases of diabetic cat and over 90% of human non-insulin dependent DM Type-2. Acute pancreatic necrosis (APN) occurs due to predisposing factors such as insulin antagonism, insulin resistance, alteration in glucose tolerance, obesity, hyperadrenocorticism, and persistent usage of glucocorticoids, as these play a vital role in the progression of APN. To manage such conditions, it is important to deal with the etiological agent, risk factors, diagnosis of diabetes, and hormonal and drug interaction along with its termination with suitable therapy (herbal) protocols. It should be noted that the protocols used for the diagnosis and treatment of human DM are not appropriate for animals. Further investigations regarding diabetic conditions of pets and wild animals are required, which will benefit the health status of all animals health worldwide.

## Introduction

Diabetes mellitus (DM) is a chronic metabolic disorder in which the body’s ability to produce or respond to the hormone insulin is impaired, resulting in abnormal metabolism of carbohydrates and elevated levels of glucose in the blood and urine. DM is one of the common and prominent metabolic diseases that have been diagnosed in canine and feline family after human beings. The clinical features described and investigated are rarely observed in other domestic large animals such as horse, cattle, buffalo, swine, and other small ruminants [1-3]. The propagation and mechanism of DM are almost similar in humans as well as animals, and thus, small laboratory animals are used in the etiopathogenesis, clinical trials, and research studies [4]. The main clinical feature of DM is thought to be the failure of β-cells to produce sufficient insulin for the metabolic pathway of the body organisms. The deceptive onset of DM depends on different factors: (a) Decline in the synthesis of insulin, (b) reduction in the sensitivity of the target cells or organs to the insulin, and (c) the excessive synthesis of other dependable hormones and drugs those are responsible for inducing DM [5].

The classification of diabetes differs for the large and small animals, although it has similarity to human. The common and general forms of DM are known to be insulin-dependentDM (IDDM) Type-1 and non-IDDM (NIDDM) Type-2 in animals. Also, the secondary DM or Type-3 has also been identified, which is the complication of the insulin antagonisms. This occurs due to the pancreatic islet damage by the pancreatic necrosis, tumor progression, and pancreatitis. Metabolic DM is the specific experimental expression of this form, primarily described in dogs and cats [2].

Clinical signs of non-ketoacidotic DM include polydipsia, polyuria, and polyphagia. In general, increased glycogenolysis and gluconeogenesis from amino acid source and little cellular uptake of the glucose trigger hyperglycemia in animals, which lasts for sometimes. Investigations have shown an association between the reduction of the glucose oxidation and such metabolic conditions. In addition, weight loss and atrophy of muscles are also associated with the abnormal gluconeogenesis of amino acid sources. Reduction in adipocyte fatty acid and increase in lipolysis will raise serum lipids, so huge amount of mobilized lipids will aggregate in the liver, which cannot be utilized or converted into lipoproteins. As a result, the liver gets enlarged uniformly with the condition of hepatomegaly. Ultimately, compensatory polydipsia, polyuria, osmotic diuresis, and glycosuria would happen due to the prolonged hyperglycemia and produce insistent high levels of glucose in urine. This will also exceed the threshold level of glucose restoration in renal tubules. Along with constant hyperglycemia, the failure of neurons in hypothalamic satiety and appetite center to uptake glucose will trigger appetite in animals [6].

This review aimed to elaborate the possible classification, etiology, and pathogenesis of spontaneous DM in small and wild animals, and also, focus on the innovative targeted researches in this area.

## Bibliographic Search

Bibliographic search for the current review was carried out through PubMed, Scopus, and Google Scholar for articles on spontaneous DM in small and wild animals. The term included “DM in canine,” “DM in feline,” “amyloidosis,” “pancreatitis,” “classification of DM in small animals,” “spontaneous diabetes in domestic animals,” “spontaneous amyloidosis in wild animals,” “glucose intolerance,” “hyperinsulinemia in animals,” and “insulin resistance in laboratory animals.”

**Figure-1:**
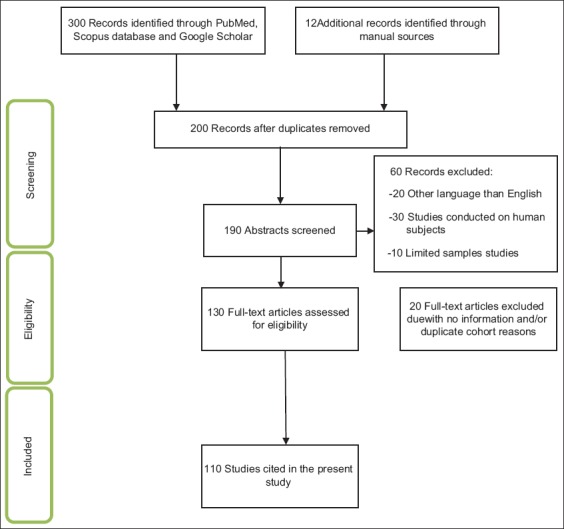
Flowchart of included studies; illustrates the number of citations and resource materials that have been screened, excluded, and/or included in this review.

The retrieved data in this article were focused on studies related to small and some wild animals. The exclusion and inclusion criteria for the present search strategy are shown in Figure-1. The articles that provided data on DM from different countries were also hand searched, and those with internet base were collected using Google as a search engine. Therefore, the total number of studies (n) included in this review was 110 (Figure-1).

## IDDM Type-1 Diabetes in Dogs

The IDDM Type-1 was first time investigated in 1861 in impulsive cases of dogs [1]. Next studies showed that this disorder particularly appears in females and old dogs even more complicated and enlarged, shortly after estrus period in pregnant females [1,7,8]. In this manner, progesterone production, which induces mammary growth hormone (GH), plays a key role as it finally triggers DM in canine [9]. During the past decade, researchers focused on the etiological elements in dogs such as Dog leukocyte antigen (DLA) that promotes DM, responsible genes, autoantibodies, and their relationships [1,10].

It has been revealed in the studies conducted on the dogs <12 months of age that concurrent occurrence of adolescent disease is an unusual event [11,12]. Besides, more than 70% of diagnosed female dogs cases suggest that they are highly predisposed to DM [7]. Vice versa, some other studies claim that DM occurs equally in males and females [13]. Surveys demonstrated that ovariohysterectomy in the yearling stage seems to eliminate DM, as it will balance or remove the hormones that are responsible for triggering diabetes commencement. It also elucidates that the incidence of DM is high in pregnant female dogs and estrous cycle.

Table-1 [7,13,14] provides various epidemiological studies conducted in the UK, North America, and Sweden, which have explored DM mainly in the different breeds of dog (e.g., Dachshund, Beagle, Lhasa Apso, Yorkshire terrier, Spitz, and Labrador retriever). Islet hypoplasia is known as the predominant and common problem in Golden Retriever and Keeshond in their first age of life. The most popular and widespread breeds such as German Shepherd and Boxer appear to be tough against DM. Usually, the DM in canine can be clinically identified at the age of 4-18 years, whereas the best average age is 7-9 years providing authentic results [7,13,14].

**Table-1:** DM commonly occurred in different breeds of dogs.

Countries	Breeds	References
UK study	Samoyed, Tibetan Terrier, and Cairn Terrier	[13]
North America study	Miniature Schnauzer, Miniature Poodle, Bichon Frise, Samoyed, and Cairn Terrier	[14]
Swedish study	Australian and Samoyed Terrier, Swedish Lapphund, Swedish Elkhound, and Border Collie	[7]

DM=Diabetes mellitus

The general damages of islets of Langerhans almost have seen in all kind of dogs. Approximately 50% of recent identified dog cases suggested that β-cell damage may be due to autoantibodies [15]. Comparing with IDDM in cattle and humans some diabetic dog shows serological reactivity to 65 kDa isoform of glutamic acid decarboxylase (GAD) or insulinoma antigen-2 [13,16]. The genes responsible to initiate DM in canine and humans such as tumor necrosis factor-alpha (TNF-α), TNF-γ, interleukin-4 (IL-4), IL-10, IL-6, IL-12β, insulin, and protein tyrosine phosphatase non-receptor Type 22 and their defensive association links have been described [1]. DLA is also known as canine major histocompatibility complex gene that acts effectively in the initiation and triggering of canine DM. Dog breeds such as Tibetan Terrier, Cairn Terrier and Samoyed explicit haplotype of major histocompatibility complex, DLA DRB1*009/DQA1*001/DQB1*008 matching with non-diabetic breeds or resistant breeds [1]. It has been revealed previously that the IDDM occurs in the Keeshonds breeds due to the presence of an autosomal recessive gene [17,18].

**Figure-2:**
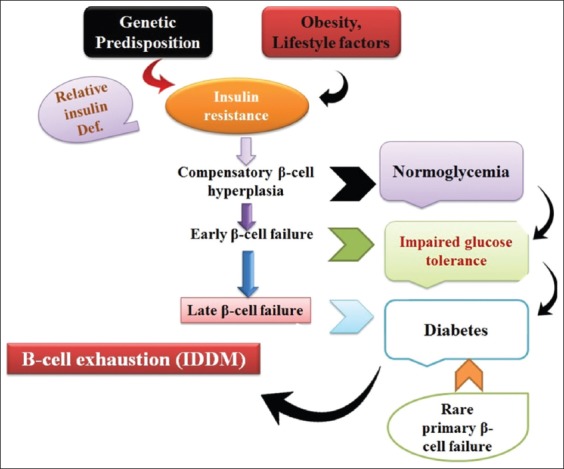
Simultaneous occurrence of insulin-dependent diabetes mellitus (IDDM) and non-IDDM in animals. Source: Designed by authors FK and FIH.

In most of the diagnostic cases in canine etiopathogenesis of DM, this is still uncertain. The appearance of DM in canine happens through different routes and due to many risk factors; therefore, the classification of diabetes in canine looks to be challenging. Inappropriately, the human classification of DM is found difficult to apply in dogs, since NIDDM Type-2 in dog shows uncertain and needs administration of insulin earlier or late in nearly all diabetic dogs. Furthermore, it was recommended that DM characterization in dogs depend on the pathogenesis rather than the response to insulin therapy [1]. The present diagnosis and therapy consider a classification system of DM in canine are primarily based on the underlying cause of hyperglycemia; insulin resistance diabetes and insulin deficiency induce diabetes (Table-2). Importantly, insulin resistance diabetes would take over insulin deficiency diabetes based on β-cell fatigue and glucotoxicity (Figure-2) [1].

**Table-2:** Characterization of DM in dogs.

Type of diabetes	Effects
Insulin deficiency, diabetes (damage of βcells and complete lack of insulin)	Hypoplasia of βcells, immunity facilitating βcell damage, βcell loss due to pancreatitis and necrosis, idiopathic processes
Insulinresistant diabetes (comparative insulin deficiency due to insulin antagonists or simultaneous conditions)	Obesity, estrous cycle, pregnancy, endocrine disorders (acromegaly and hyperadrenocorticism), glucocorticoids, and synthetic progestin

DM=Diabetes mellitus

In some of the dog breeds such as Golden Retriever, Dachshund, and Shetland Sheepdog, obesity and DM have an imperative association; as in fully obese dogs, there will be unusual glucose xenophobia and high level of insulin [19,20]. Later on, it was determined that DM in the obese dogs could be classified on the basis of the reaction to the glucose administration. In the first group with abstaining hyperinsulinemia, there was a worthy response to the glucose level by increasing insulin secretion, and in the second group, no response to the glucose administration was highlighted by fasting excessive insulin in the blood. These results indicated that there was a low level of insulin in obese diabetic dogs as compared to obese non-diabetic dogs [21]. Lai *et al*. [22] reported that high-fat foods trigger successive insulin resistance which is not followed by excessive insulin in the circulatory system and ultimately lead to DM and glucose intolerance. Despite information above, DM initiated by insulin resistance and high insulin level in dogs is considered as an uncommon condition. Occasionally, persistent corpus luteal and pseudopregnancy in female dogs generates gestational DM (GDM); furthermore, the extensive use of artificial gestagens such as megestrol provokes DM by vacuoles formation in the β-cells [23].

Very few studies stated that GDM mostly occurs during the estrous cycle in dogs [7,8,24]. During fetus development, vital metabolic changes happen to assist the new developing fetus. Throughout the pregnancy, there would be an increased level of lipid metabolism, high insulin secretion, higher energy diet, and peripheral insulin resistance, and accordingly, these metabolic changes will offer the energy in the form of amino acids and glucose for newborn growth. The surplus lipids already stored in the adipose tissue are being used as an alternate source for female maintenance and survival. These efficient functional and morphological changes also induce hyperplasia and hypertrophy in the islets of Langerhans β-cells and activate insulinemia, which sustains normal glycemia ultimately [25]. The failure of the adaptive mechanism leading to GDM in canine, as previous findings, proved that hormones such as lactogens, prolactin, cortisol, and progesterone can generate peripheral insulin resistance. Progesterone plays an important role in the development of GDM as initiates’ mother GH and prompts anti-insulin activity [26]. In general, glucose toxicity occurs due to the lack of insulin and damage of the β-cells. Higher levels of GH and cessation of GAD-65 autoantibodies present during diestrous phase in pregnancy and pseudopregnancy [8]. Verdicts of above studies reveal that reproductive hormones are also crucial for the initiation of DM, and likewise, surgical procedure as ovariohysterectomy and termination of pregnancy gives good extensive enhancement [8]. Note that, almost more than 50% of affected animals would normalize the following procedures. Together, these investigations demonstrated in aged animals, and DM will be a permanent condition due to the reduction and exhaustion of β-cell ooze as well as the declined time of tolerance to hyperglycemia.

## NIDDM Type-2 Diabetes in Cats

**Figure-3:**
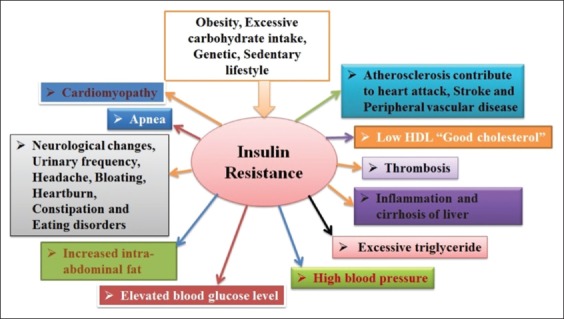
Complication occurred due to diabetes mellitus in animals. Source: Designed by authors FK and FIH.

In cats, NIDDM most frequently emanates post-pathological conditions encountered by endocrine system disruption [27]. DM in cats shows the same clinical and pathological characteristic as human Type-2 diabetes restricted to individual characteristics such as obesity, median, and older age, following lower blood insulin level or the aggregation of amyloids in islets of Langerhans with damaged β-cells, and finally lead to retina and neural complications (Figure-3). The administration of drugs/hormones such as corticosteroids, progesterone as well as various diseases such as acromegaly, hyperthyroidism, renal, cardiac conditions, and hypercortisolism trigger insulin resistance in cats. Also, cats express extra insulin dependency and ketoacidosis [28-31]. IDDM exploration seems problematic in cats: As β-cell antibodies did not prove any involvement in the pathogenesis of diabetes in cats, besides the rare inflammation of lymphocytic islets of Langerhans [32,33].

In cats, secretion of insulin decreases as a response to glucose during the first phase of DM, following an exaggerated response in the second phase at early stages of the disease. Induction of glucotoxicity, lipid toxicity, and the islets of Langerhans amyloid disease strongly are involved in the initiation and development of DM, especially NIDDM Type-2 in cats. The first confirmatory model in cats exhibited the harmful effects of glucose on islets of Langerhans after long therapy of intraperitoneal administration of glucose to form vacuole in Langerhans cells that have led to DM. The structural and functional damages of β-cells and target tissue of insulin could be the main aim of therapy protocol of hyperglycemia to protect β-cells and lessening of DM. Glucose is responsible for the initiation of insulin synthesis through simulating dictation of insulin gene by phosphorylation of pancreatic and duodenal homeobox 1 (PDX1). It has been reported that glucotoxicity and hyperglycemia initiation might appear due to the reduction of insulin, PDX1, and β-cell deregulation of glucose transporters on membrane. Early death and exhaustion of β-cells were observed as the result of the endurance of hyperglycemia for more than 10 days. Extraordinary oozes of insulin and β-cell damages elucidate glucotoxicity [34,35].

Reusch [31] showed basal insulin resistance, and the initial stage of DM happens through the interaction between excessive reaction to glucose and hyperinsulinemia. Amyloids degradation of islets of Langerhans and debilitation of β-cells are typically supplemented by remunerative insulin production and secretion failure, which leads to explicit DM. In addition, early stages of the disease in cats did not need insulin, while later on, it becomes a compulsory component [31]. There are significant values regarding the incidence of DM in certain population like 0.43-2.24% substantially in Burmese cats. Comparing the sex and age of cats, DM is prominent in male and significantly more advanced in older (>13 years of age) associating with long-haired and small pet breeds [36-38]. DM in adolescence was infrequently characterized. The most diagnostic features such as hypoplasia of islets of Langerhans and blurred vision due to the diabetic bilateral cataract involving cortex and nucleus also help to classify DM in cats [12]. The symptoms of occurrence are similar with human’s diabetes; the diabetic cats persisted amyloid disease, damage to α-and β-cells, while normal δ-cells. However, these disorders do not impair glucose tolerance, and still, it has some vital influence in the pathogenesis of DM [33].

In domesticated and wild cats, more than 80% identified cases of NIDDM showed islets of Langerhans amyloid disease typical appearance [38,39]. The quantity of amyloid protein aggregation increases with age and typically identified in normal and healthy cats as well. The amyloid disease condition is much pronounced in diabetic individuals as compared to the non-diabetic one. The existences of high levels of amyloid protein lead to the devastation of β-cells, diminishing of β-cell activity, and high stimulation of β-cells. Aggregation of amyloid in extra- and intra-cellular space in non-endocrine cells of islets of Langerhans causes cellular death and additional cellular membrane interruption [40]. The by-products of amylin peptide hormone cosecreted with insulin from β-cells are predicted to impair glucose tolerance. Amylin also known as islet amyloid polypeptide (IAPP) inhibits glucagon secretion and deficient in diabetic patients [30,41,42]. The concentration of IAPP has been reported to be high in hyperinsulinemic clamp and obese cats with diminished glucose resistance. Not only a high concentration of IAPP is compulsory for islet amyloid protein formation but also other mechanisms such as malformation of β-cells, emission, carrying, and deprivation of IAPP may also be involved [43]. A robust association between IAPP and initiation of Type-2 diabetes were detected in both humans and cats. Likewise, the initiation of DM in cats is accompanied due to the obesity in correlation with high concentrations of IAPP and insulin in non-diabetic individuals [44]. Findings suggest that hyperglycemia also happens due to the inhibitory effect of amylin hormone on glucose-exciting insulin formation, which at last enhances gluconeogenesis. Glycogenolysis and reservation of glucose intake develop regarding IAPP persuaded insulin conflict in muscle. It was assumed that secondary obesity occurs when glucose yields from muscle glycogenolysis, which has been recycled in the process of conversion of acetyl-CoA to fatty acid as IAPP, do not affect fat tissue [45].

Aggregation of non-adipose tissue around the heart, liver, pancreas, and skeletal muscle along with unnecessary high levels of triacylglycerol (TAG) initiates lipid toxicity. Moreover, insulin resistance takes place due to the excessive accumulation of TAG in β-cells, which will exhaust these cells and alter insulin synthesis. The existence of TAG in these cells could be insufficient to persuade small quantity of insulin and insulin resistance. It is understood that fatty acids are generating peroxidation compounds and toxic lipid intermediates due to the production of TAG and hydrolysis of triglycerides along with lipolysis. In addition, the alteration in insulin signaling of β-cells and liver initiated by TAG, and toxic lipid intermediates substantially promote the expression of the genes responsible for death. Li *et al*. [46] have indicated that in non-adipose tissue, insulin resistance and lipid toxicity are initiated by vital long-chain 3-hydroxy acyl-coenzyme-A dehydrogenase (LCHAD). Similar mechanism involves in skeletal muscle. They also reported that low intracellular glucose concentration usually correlates with higher levels of LCHAD in sarcoplasm. Furthermore, insulin resistant in humans and animal individuals shows high intramuscular quantity of LCHAD [46].

Mass of adipose tissue is supposed to be associated with the effectiveness of insulin to lessen basal glucose concentration. Male cats with heavyweight have a failure in insulin sensitivity, while commonly lean male cats have less insulin delicate than lean female cats. Obese male cat develops hyperinsulinemia faster than obese female individuals, and high insulin resistance can be observed in high abdominal fat aggregation. It has been explored that cats’ breed such as Burmese has excessive abdominal fats accumulation and low subcutaneously [37]. Moreover, not only obesity results in DM but also the onset of diabetes involves smaller improvement in body weight gain and adipocytes increase. Statistics suggested that 25% of cases showed insulin sensitivity due to the slight weight gain as compared with diabetic individuals [47]. Alteration of internal oxidative glucose metabolism and less compatibility of insulin receptors leads to more insulin resistance.

As described earlier, low level of glycogen in liver cell cytoplasm and sarcoplasm leads to the higher insulin resistance. In addition, IAPP inhibits glycogen synthetase and glucose uptake and increases glycogenolysis followed by lactate synthesis. Subsequently, gluconeogenesis is begun soon after liver uptakes lactate. So far, three main insulin signaling genes are recognized as key elements in liver and muscle of moderate obese cats: (1) Phosphatidylinositol 3 kinases p-85-α, (2) insulin receptor substrate-1 (IRS-1), and (3) IRS-2. The incidence of insulin resistance in NIDDM showed similarity in both feline and humans [48].

**Figure-4:**
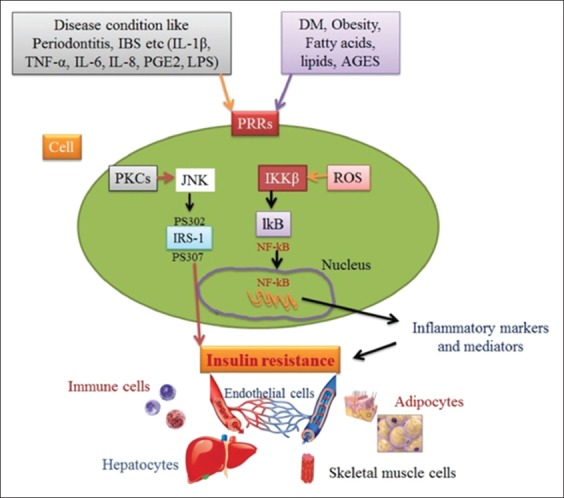
Mechanism initiating insulin resistance. Source: Designed by authors FK and FIH.

Alteration in glucose tolerance and insulin resistance peruses a significant influence on NIDDM, such as obesity [49]. Obesity and compensatory excessive circulatory insulin trigger low tissue sensitivity to insulin. Figure-2 depicts multifactorial causes of obesity such as genetics, nutrition, and environment. Rand *et al*. [50] have reported that the lack of nutrition leads to lipid degradation and excessive food toxicants, which as a result initiates lipid formation based on a genetic factor in the existence of energetic thrifty genotype. In addition, consumption of an excessive amount of edible diet with extra supplementation of carbohydrates, aging, castration, vasectomy, hysterectomy, and the little physical workout is also counted as important factors [51]. Higher concentrations of IL-1β, IL-6, C-reactive receptors, leptin, resistin, and TNF-α indicate the initiation of obesity and insulin resistance (Figure-4). It has been stated that mainly, insulin receptors that have been blocked by TNF-α would affect insulin sensitivity [49].

## DM in Wild Animals

Concurrent occurrence of DM in humanized and non-humanized primates was identified in confined animals. Clinical and morphological characteristics in restricted animals explored as NIDDM Type-2. Various studies suggest that insulin resistance causes alteration of β-cell activity and prolong pre-diabetic conditions and thus also has shown resemblances of NIDDM in both humans and non-human primates. As yet, synchronized amyloid disease has been identified in different animals: *Pan troglodytes* (chimpanzee), *Pongo pygmaeus* (orangutan), *Cercopithecus* species, *Macaca* species, *Mandrillus* species, *Papio hamadryas, Saimiri sciureus*, and *Macaca mulatta* [52-58]. Aggregation of amyloid protein has been identified in different organs such as in the liver, kidney, pancreas, spleen, and adrenal gland. In research by Hubbard *et al*. [57], immunohistochemical analysis of IAPP, calcitonin family peptide, and calcitonin gene-related peptide showed positive aspects of islets of Langerhans amyloid disease. Histopathological lesion assays showed that amyloid protein accumulation throughout capillaries and in pancreas cells has serious harmful effects that were similar to those defined in cats [53,57,59].

Fibrosis of islets of Langerhans and chronic pancreatitis was identified in *Procavia capensis* (rock hyrax) and California sea lion, describing another type such as secondary DM [60,61]. *Panthera onca*, African spotted leopard, and even their progeny developed DM in confined conditions [62,63]. Consequently, the extensive use of humanized hormone-megestrol acetate to prevent pregnancy and following subtype of breast cancer leads to elucidate secondary DM in *Panthera onca* [62].

DM in older primates causes disorders such as venous thrombosis, nephropathy, myocardial fibrosis, and cardiomyopathy [64]. Non-humanized primates such as monkey initiate pituitary adenoma, which is a favorable situation to insulin antagonism leading to secondary DM [65]. The study of *Spermophilus lateralis* was one of the comprehensive and widely investigations that exhibited the presence of DM in confined rodents. Obviously, these animals are mostly characterized by blurred vision, retinal atrophy, and opacity, plus the vacuole formation has been observed in their islets of Langerhans in both α-and β-cells [66]. Rodents such as *Ctenomys talarum* and *Lagostomus maximus* provoked DM in progeny as well as in adults with noteworthy high levels of fructosamine, glucose, fatty liver, opacity, and blurred vision [67,68]. It is hypothesized that diminishing exercise, excessive diet, and more glucose consumption appear to be other reasons for DM as well. It seems that a few months of confinement elucidated IDDM Type-1 in individuals and their progeny such as in *Clethrionomys glareolus*. Certain animal studies have illustrated that IDDM shows specific indications, such as islets of Langerhans vacuole formation, and insulin autoantibodies, such as islet antigen-2 and GAD-65. In the pancreatic islets, vacuolization *picornavirus* was also recognized [69,70].

The granivores bird predators have different strategies for the metabolism of glucose as compared with other mammals. Although there were high concentrations of glucose in birds as compared with other vertebrates matching body weight, lower intracellular glycogen and plasma glucose level were not controlled by insulin involvement. Three various types of islets of Langerhans are responsible for the synthesis, production, and secretion of the avian pancreatic polypeptide, somatostatin, insulin, and glucagon [71]. The glucagon concentration in birds is greater than mammals, due to its activities toward glucose metabolism as well. The well-organized mechanisms, which make birds urine free of glucose, also need glucose transport protein and sodium-glucose co-transporters to absorb glucose from gastrointestinal tract [72]. Perhaps, in conclusion, it may be suggested that lower insulin and higher glucagon are significant for provoking DM [71]. Another study in ducks proposed that surgical removal of pancreas express hypoglycemia. Indeed, there were controversies as administration of anti-insulin serum, and partial surgical removal of pancreas elucidates DM in ducks and gooses. Furthermore, in few cases of birds, DM was fruitfully achieved by long-term insulin therapy [73].

Van Der Kolk *et al*. [74] exhibited in bulls which have chronic laminitis, polyarthritis, and traumatic injuries of the penis with edema treated by chlorothiazide, dexamethasone, phenylbutazone, and prednisolone expressed islets of Langerhans atrophy and fibrosis. The authors stated that *Elephantid herpesvirus-1* could be the possible source of this condition and treatment procedure should also contain the essential factor such as the use of insulin antagonist. Nevertheless, *Psittacid herpesvirus-1* in the pancreas was infrequently found, but differential diagnosis of bird pancreatitis with or without DM is important. Interestingly, a significant linkage between DM and extreme iron deposition was monitored in *Ara severa, Ara militaris*, and humans [75]. Studies of iron overloading revealed the presence of insulin insufficiency related to deposition of iron in β-cells in humans and rodent [76]. In a dispute, no prominent lesions were found in islets of Langerhans. Still, long-term protocol flourished birds survival for about 1.5 years [75].

In raptors, DM is rarely reported, while in carnivorous birds such as a great horned owl, surgical pancreatectomy resulted in hyperglycemia and consequently caused death. Concurrent DM was also identified in female *Buteo jamaicensis*, where the main lesions such as air sacculitis, bronchopneumonia, and cysts are formed in proximal tubules linked with interstitial lymphocyte nephritis. Although individual foci were identified in fatty liver syndrome, the absence of typical glycogen granules and β-cells was limited with severe deterioration of islets of Langerhans. However, both histological and bacteriological examination revealed cocci in the lungs and kidneys with no bacterial clusters, respectively. Hence, the etiology lasted mysteriously, and no tumor in pituitary and adrenal glands was observed [77].

The clinical diagnostic lesions such as ketonuria, renal glucosuria, hypoinsulinemia, hyperglycemia, polydipsia, and polyuria were found infrequently in species such as in *Ara severa* and *Nandayus nenday* [78,79]. The absence of immune histochemical reaction to insulin nearly damages all islets of Langerhans. Almost in the entire conditions of exocrine pancreas, clinical autoimmune pancreatitis was identified [78]. The islets of Langerhans hypertrophy and remunerative proliferation were investigated in *Psittacus erithacus*, in which the damage was elucidated by pancreatic inflammation [80]. Bacteria and viruses were also found in simultaneous cases, such as Pacheco’s disease caused by *Psittacid herpesvirus* that is presented in lymphoplasmacytic pancreatitis in *Nymphicus hollandicus* diagnosed with IDDM [81]. The analysis showed the similarity of this disorder with *Psittacines* and was highlighted in intestinal crypts, parathyroid, thymus, nuclear inclusions, cytoplasmic inclusions, syncytial cells in respiratory epithelium, and hepatic/renal and splenic necrosis [82,83]. Table-3 provides an overview of the existing animal models, which are used to evaluate different types of DM in animals.

**Table-3:** Existing animal models for the assessment of DM.

Animal model	Type of animals used	Experimental parameters
Normoglycemic	Normal healthy animals	Potential oral hypoglycemic agents are tested
Oral glucose loading model (glucose tolerance testing)	Normal healthy animals	Physiological induction of DM in animals; glucose level is monitored
Streptozotocin model	Normal healthy animals	Streptozotocin induces DM over a period; provides suitable time intervals and additional information for the mechanism of action of drugs
Alloxan model	Normal healthy animals	Alloxan induces DM; provides the possibility to produce different grades of severity of DM; provides the blood sugar levels equivalent to total pancreatectomy
Ferric nitrilotriacetate induced DM model	Normal healthy animals	Measures diabetic symptoms(hyperglycemia, glycosuria, ketonemia, and ketonuria)
Surgical model	Normal healthy animals	DM is induced in removed pancreas(pancreatectomy); permits to assess if the drug affects resistance to and secretion ofinsulin
Genetic model	Genetically engineered animals or mutant strains	Longterm complications of DM could be monitored; several drugs could be tested against DM; measures(blood sugar, body weight, insulin production, and insulin resistant); explores the mechanism of action of drugs

DM=Diabetes mellitus

## Diabetes and Pancreatic Disease

The characteristic lesions of chronic pancreatitis include fibrosis, cyst formation in pancreatic ducts, lymphocytic penetration, and atrophy of parenchyma [84]. Simultaneous slight incidents of acute pancreatic necrosis (APN) and acute pancreatitis provoke chronic inflammation. The initiation of DM in dogs, cats, and other wild animals due to the chronic pancreatitis is not always responsible for inducing degeneration of pancreatic islets. The endocrine and exocrine deficiencies may develop over the excessive fibrosis, trophy, and lympho-plasmatic penetration in the pancreas. However, this is also noteworthy that DM occurrence related to chronic pancreatitis has been reported in nearly 30% of identified cases in association with histological characteristic [85]. Breeds’ predispositions exhibited a positive association with the incidence of DM, while old-aged dogs and cats demonstrated wide damages of parenchyma and secondary DM regulated by different morphological forms of adenocarcinoma of the pancreas [3,86].

The degeneration of islets of dogs and cats through the pancreatic necrosis is followed by the acute and chronic pancreatitis and tumor formation. Primarily, the dominance of tumor and inflammation encloses canine APN and acute pancreatitis. The term APN is used for tumor, is reliable, and thus is signified with inflammation. APN rarely occurs in cats, is sporadic in pigs, and deliberately is considered as the main reason of DM in dogs. Therefore, the initiation of DM is regulated by various influencing features of APN. Predisposing factors such as insulin antagonism, insulin resistance, glucose tolerance alteration, obesity, hyperadrenocorticism, and persistent use of glucocorticoids are also associated with APN. Further, along with these, other complications take place if DM is not properly noted and treated (Figure-3). Acute inflammation of the pancreas expresses diabetic ketoacidosis, whereas ketonuria exists as fatal APN in most of the dogs. Typically, acute pancreatitis and ketonuria along with APN develop DM so that pancreatitis may progress due to the DM. Hypertriglyceridemia condition in diabetic dogs presents as threat feature for pancreatitis [87,88].

Tumors of islets of Langerhans are rarely diagnosed in dogs. Pancreatic tumors cause secretion of the excess amount of hormones, as most of the diagnosed dogs and ferrets exhibited immoderate levels of hormones in histological examination. DM is also related with glucagonoma of the pancreas and is included by antagonism of glucagon through insulin. Both mechanisms of hepatic glycogenolysis and gluconeogenesis are responsible to elucidate hyperglycemia and extreme secretion of glucagon [3,89].

## Non-ketotic Hyperosmolar Diabetes (NKHOD) and Ketoacidosis Diabetes

Hyperglycemia, osmotic diuresis, and hyperosmolarity followed by dehydration are termed NKHOD. Accordingly, coma, hyperthermia, seizures, ataxia, convulsions, and nystagmus are the deleterious clinical features of the central nervous system [90]. The most prominent and severe complication of DM in dog and cats is ketoacidosis. The elevation of blood insulin antagonists such as cortisol, glucagon, and monoamines, along with the enhancement of hormones, generates stressful conditions due to the poor management of insulin treatment. Additional disorders such as pancreatitis, necrosis, infection, Cushing syndrome, immune suppression, renal, hepatic, and heart failure trigger simultaneous DM. Such conditions initiate lipolysis and make liver to accumulate more fatty acids regarding survival and body maintenance. Acetoacetic and β-hydroxybutyric acids are formed due to deficiency of insulin and glucose, which in turn facilitate oxidation of triglycerides. Certain ketoacids are modified into acetones and exhale air contained acetones and ketones in the breathing air [91]. Clinical features such as hypovolemic shock, dehydration, and excessive loss of magnesium, sodium, potassium as well as conditions like ketonemia, ketonuria, acidosis, and hyperglycemia provoke ketoacidosis [92].

## Hormones Associated with DM and Drugs

Acromegaly is expressed as the hormonal disorder, in which too much GH is secreted, thus initiates significance adenoma of pituitary gland, linked with severe insulin resistance and NIDDM Type-2 [93]. The higher levels of GH will trigger the extra production of insulin growth factor-1 (IGH-1), which exerts anabolic effects and subsequent propagation of bones, organs, cartilage, and tissues. The level of IGH-1 in cats determines the indication of acromegaly with definite insulin resistance that is why both GH and IGH-1 are considered for diagnosis [94,95]. The body demands of glucose are high due to hyperthyroidism, which explicates extra triiodothyronine (T_3_) and thyroxine. Therefore, extra glucose is synthesized by natural process in the liver through lactic acid cycle and gluconeogenesis. Furthermore, this extra fatty acids and glycerol are also produced from lipolysis of adipose tissues in fasting time. The main resources of glycerol and amino acid are known to be lipolysis and proteolysis, respectively, based on the initiation of gluconeogenesis in certain conditions. Excessive anaerobic glycolysis and reduced insulin-stimulated glycogen create hyperthyroidism conditions. In result, the adipose tissues are reserved and hyperglycemia is maintained due to the anaerobic glycolysis of muscles, which produce lactate and convert to the glucose in liver [96].

Hormones and drugs which disturb insulin synthesis and activity play a crucial role in the induction of DM in animals. Hence, GF, thyroxine, glucagon, adrenaline, glucocorticoids, progestin, and artificial progesterone are mostly elaborated. Antagonist action is usually elicited on peripheral tissues, followed by collapse, damage to islets, and sustained hyperglycemia. Ultimately, if antagonism is no further organized in individuals, then animals will promote to IDDM condition. In general, necrosis is triggered by excessive adrenocorticism, whereas administration of dexamethasone and prednisolone represents antagonism in dogs, but these drugs hardly create hormonal abnormality in cats with simultaneous insulin resistance [97-99]. Structural and functional changes to epithelial tissues, especially in adrenal and pituitary glands, initiate hyperadrenocorticism in more than 50% of diagnosed cases affected by diabetes. Complete control of glucose in the blood (glycemia) was rarely established and diagnosed by typical signs.

Insulin antagonism linked with the high secretion of monoamines such as catecholamine in an animal with pheochromocytomas. Monoamines act as insulin inhibitors and produce a mild condition of intermediate glucose intolerance [100]. Diuretic drugs such as furosemide and hydrochlorothiazide induce glucose intolerance by the reduction of insulin-stimulated transport in adipose tissues and skeletal muscles [101].

## Herbal Therapies in the Treatment of Diabetes

Herbal therapies in diabetes cover a wide range of studies and investigations. Mirhoseini *et al*. [102] published a paper, which introduced 27 medicinal plants used in animal models for the treatment of DM. Silymarin a well-known phytochemical (a standardized extract of the seeds of *Silybum marianum*) is believed to lessen inflammatory cytokines and oxidative stress biomarkers to prevent the IDDM type-1 in patients [103]. Herbal therapeutic agents such as Setarud (IMOD™ have the potential to reverse diabetes in streptozotocin-induced diabetic models [104]. Today, the utilization of herbal agents is passing a growing route in Asia, Europe, and North Africa as a tonic, anti-diabetic, anti-pyretic, and anti-allergic. *Phlomis* species such as *Phlomis persica* boiss or its extract significantly downregulate blood glucose level and are also effective against the complication that occurs due to the lipid peroxidation by antioxidant activity in streptozotocin-induced rats [105]. Similar investigation exhibited that *Bixa orellana* (annatto) extract decreased blood glucose levels in fasting normoglycaemic and streptozotocin-induced diabetic dogs by stimulating peripheral utilization of glucose [106]. The uses of conventional antidiabetic drugs along with herbal remedies have shown possible antihyperglycemic and therapeutic adjuncts of the oral route for controlling diabetes [107]. The therapies such as Setarud in combination with other polyphenols or flavonoids such as curcumin and quercetin have much stronger antioxidant effects in reversion of diabetes, yet preclinical and clinical trials should be elucidated for exact mechanism [108]. Besides herbal agents, other trace elements such as selenium act as antioxidants; however, patients with diabetes express an increased level of oxidative stress along with deficient selenium. Selenium exerts adverse effects on blood glucose hemostasis [109]. Bahadar *et al*. [110] reported that the massive exposure of arsenic and other environmental intoxication and pesticides play a significant role in the incidence of diabetes in humans as well as in animals, which is still unnoticed in non-social animals.

## Conclusion

The spontaneous DM in small and wild animals comprises all types of diabetes defined in humans. In dogs, pregnancy, obesity, diestrus phase, and obstinate corpus luteum may generate diabetes. The appearance of DM in wild animals can be correlated with different stimuli such as confinement, food changes, low physical exercises, pancreatitis, and hormonal imbalance in the body. In cats, spontaneous NIDDM related to the islets of Langerhans amyloid disease is considered as the proper animal model but infrequently identified. Hormones, drugs, and pancreatic ketoacidosis collate to induce diabetes. To date, several herbal therapeutic agents with remedial potential are known to treat diabetes in humans and should be introduced in small and laboratory animals as well.

DM in animal models is not only a substantial area of research due to the various similarities with humans but also will play a key role in veterinary medicine and its progression. The investigation and the establishment of therapeutic protocols for diabetic animals comprise different issues. This should start with the recognition of etiological and risk factors in association with diabetes diagnosis of the DM initiators and suitable treatment protocols to terminate DM, in combination with other herbal therapies. This is noteworthy that human diagnosis, treatment, and classification protocol are not appropriate for animals, and the concurrent onset of DM in animals signifies the primary steps in creating unique and sufficient models for further studies.

## Authors’ Contributions

MA gave with the idea, KN, and FM wrote the first draft of the manuscript. FK and FIH designed the figures. SM made the tables. Then, the whole manuscript critically revised by MA. All authors have read and approved the final version of the manuscript.
